# Development of a Highly Efficient CRISPR/Cas9-Mediated Herpesvirus of Turkey-Based Vaccine against Novel Variant Infectious Bursal Disease Virus

**DOI:** 10.3390/vaccines12030226

**Published:** 2024-02-23

**Authors:** Jun-Feng Zhang, Jong-Yeol Park, Sang-Won Kim, Yu-Ri Choi, Se-Yeoun Cha, Hyung-Kwan Jang, Bai Wei, Min Kang

**Affiliations:** 1 College of Medical Technology and Engineering, Henan University of Science and Technology, Luoyang 471003, China; jfzhang018@gmail.com; 2 Department of Avian Diseases, College of Veterinary Medicine and Center for Avian Disease, Jeonbuk National University, Iksan 54596, Republic of Korea; jyp410@naver.com (J.-Y.P.); sk970221@gmail.com (S.-W.K.); 9cinderella7@naver.com (Y.-R.C.); seyeouncha@jbnu.ac.kr (S.-Y.C.); hkjang@jbnu.ac.kr (H.-K.J.); 3 Bio Disease Control (BIOD) Co., Ltd., Iksan 54596, Republic of Korea

**Keywords:** variant infectious bursal disease virus, recombinant vector vaccine, herpesvirus of turkeys, CRISPR/Cas9

## Abstract

Infectious bursal disease (IBD), caused by IBD virus (IBDV), is an extremely contagious immunosuppressive disease that causes major losses for the poultry industry worldwide. Recently, the novel variant IBDV (G2d) has been highly prevalent in Korea, but the current vaccines against this very virulent IBDV have limited efficacy against this novel variant. To develop a vaccine against this variant IBDV, a recombinant virus designated rHVT-VP2 was constructed by inserting the IBDV (G2d) VP2 gene into herpesvirus of turkeys (HVT) using CRISPR/Cas9 gene-editing technology. The PCR and sequencing results obtained showed that the recombinant virus rHVT-VP2 was successfully constructed. Vaccination with rHVT-VP2 generated IBDV-specific antibodies in specific pathogen-free chickens starting from 2 weeks post-immunization. Seven days after the challenge, the autopsy results showed that the bursa atrophy rates of the rHVT-VP2, HVT, vaccine A, and positive control groups were 0%, 100%, 60%, and 100%, respectively, and the BBIX values were 1.07 ± 0.22, 0.27 ± 0.05, 0.64 ± 0.33, and 0.32 ± 0.06, respectively. These results indicate that rHVT-VP2 can provide 100% protection against a challenge with the IBDV (G2d), whereas vaccine A only provides partial protection. In conclusion, vaccination with the recombinant virus rHVT-VP2 can provide chickens with effective protection against variant IBDV (G2d).

## 1. Introduction

Infectious bursal disease virus (IBDV) is a considerable economic threat to the poultry industry worldwide. IBDV targets and destroys the precursors of antibody-producing B cells in the bursa of Fabricius of young chickens, thus inducing immunosuppression, which leads to vaccination failures and an increased susceptibility to other infectious agents [1]. IBDV has two known serotypes (I and II); however, only serotype I is pathogenic to chickens. Serotype I is divided into four primary pathotypes based on pathogenicity and antigenicity: classical virulent (cv), antigenic variation (av), very virulent (vv), and attenuated (at) [1]. Recently, a new classification of IBDVs with seven genogroups (G1–G7) has been proposed due to the fast genetic variation in the hypervariable (hv) VP2 area, which is considered a major protective antigen that elicits neutralizing antibodies to protect chickens from IBDV infection [2,3]. The cv/atIBDV, avIBDV, and vvIBDV strains correspond to G1, G2, and G3, respectively; G4 includes the ‘distinct’ (d) IBDV strains; G5 contains strains isolated from Mexico; and G6 and G7 contain strains mainly from Italy and Australia, respectively [2,3]. G2 has been further geographically divided into the sub-lineages G2a, G2b, G2c, and G2d. Recently, studies have shown that G2d, the dominant genogroup, is highly prevalent in Asian countries, including China, Malaysia, Japan, and South Korea, and is a new threat to poultry farms [4,5,6]. Previous studies have shown that this new variant of IBDV could cause more severe pathological lesions than existing variant strains [4]. In addition, current vaccines do not provide efficient protection against this novel variant; therefore, an updated antigen-matched vaccine against G2d variant IBDV is urgently needed to protect domestic poultry [7].

Live attenuated IBDV vaccines are commonly used in the chicken industry to control IBD [1]. However, live IBDV vaccines also cause varying degrees of damage and atrophy of the bursa, and the production of attenuated vaccines is time consuming and labor-intensive; furthermore, these live vaccines are less effective in the presence of maternal antibodies [1]. In contrast, herpesvirus of turkeys (HVT) is widely used as a vaccine vector for the expression of heterologous antigens against a number of avian diseases, including IBDV [8]. HVT-associated vector vaccines are also effective in the presence of maternal antibodies and capable of providing life-long immunity. The currently available method for generating HVT recombinant vaccines is based on homologous recombination, artificial bacterial chromosomes, and fosmid systems [9,10]. However, these technologies are often time consuming and labor-intensive. Recently, proof-of-concept studies have shown that the epoch-marking genome-engineering technology clustered regularly interspaced short palindromic repeats (CRISPR)/associated 9 (Cas9) is a rapid and efficient method for generating recombinants, which has spurred the development of effective poultry vaccines [11,12,13,14].

In the present study, we took advantage of CRISPR/Cas9 to rapidly generate a recombinant HVT vector vaccine containing the VP2 gene from the IBDV G2d strain targeting the currently prevalent novel variant of IBDV. Recombinant rHVT-VP2 vaccines were further evaluated to assess the induced immune reaction and their protective efficacy against novel variant IBDV challenges in chickens.

## 2. Materials and Methods

### 2.1. Viruses and Cell Culture

The HVT Fc126 strain (GenBank accession number, AF291866.1) was stored in liquid nitrogen, and the IBDV strain A21-ETC-014 (G2d, GenBank accession number, OM310973) was identified and preserved at −70 °C in our laboratory. Primary chicken embryo fibroblasts (CEFs) from 10-day old specific pathogen-free (SPF) chicken embryos (Sunrise Farms, Inc., Stuarts Draft, VA, USA) were prepared according to methods previously described [11]. All HVT strains were propagated in primary or secondary CEFs. Isolation and titration of IBDV strains were performed in 10-day old SPF chicken embryonated eggs via the chorioallantoic membrane (CAM) route, as previously described [15]. 

### 2.2. Construction of the Cas9/gRNA Expression Plasmid and Donor Plasmid

The gRNA targeting the UL45/46 region of the HVT genome, sg-A, and donor plasmid were described previously [11]. And the gRNA was cloned into the CRISPR expression plasmid pSpCas9(BB)-2A-Puro (PX459) V2.0 (Addgene, Watertown, MA, USA) to yield PX459-UL45/46-gRNA by inserting the synthesized primers UL45/46-gRNA-F/R into *Bbs*I restriction sites [9,16]. Similarly, the bait sequence sgA with no homology to the genomes of humans, chickens, pigs, prokaryotic DNA sequences, or viruses was cloned into PX459 to yield PX459-sgA-gRNA [17].

To construct the donor plasmid pGEM-sgA-LoxP-PacI-GFP-PacI-LoxP-SfiI-VP2-SfiI-sgA containing green fluorescent protein (GFP) and IBDV (G2d) VP2 expression cassettes, sgA-LoxP-PacI-LoxP-SfiI-spacer-SfiI-sgA was synthesized using a commercial gene synthesis service (Cosmogenetech, Seoul, Republic of Korea) and cloned into pGEM-T-easy vector to generate pGEM-sgA-LoxP-PacI-LoxP-SfiI-spacer-SfiI-sgA. The GFP expression cassette from pEF-GFP (Addgene, Watertown, MA, USA) was cloned into pGEM-sgA-LoxP-PacI-LoxP-SfiI-spacer-SfiI-sgA via the PacI site to generate pGEM-sgA-LoxP-GFP-LoxP-SfiI-spacer-SfiI-sgA. The VP2 (G2d) expression cassette was then cloned into pGEM-sgA-LoxP-GFP-LoxP-SfiI-spacer-SfiI-sgA via SfiI sites to generate the final donor plasmid pGEM-sgA-LoxP-PacI-GFP-PacI-LoxP-SfiI-VP2-SfiI-sgA. The primer sequences used are listed in Table 1.

### 2.3. Generation of Recombinant rHVT-VP2

**Figure 1:**
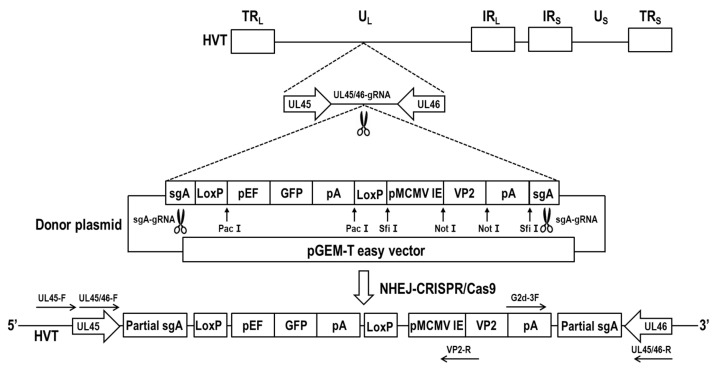
Strategy used to generate the recombinant rHVT-VP2 virus.

**Figure 2:**
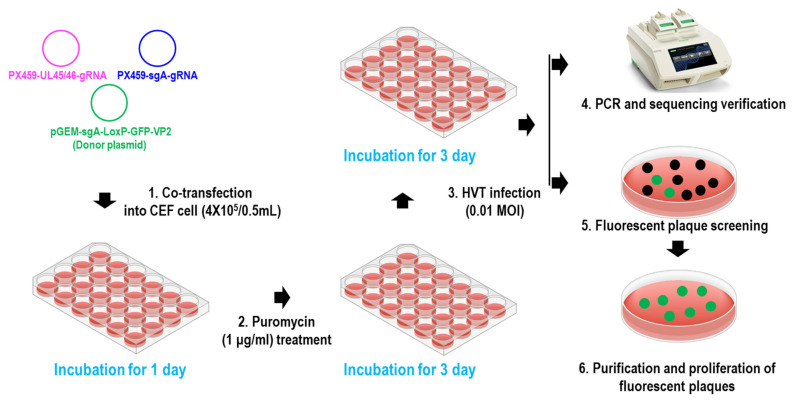
Diagram depicting the protocol used to generate the recombinant virus rHVT-VP2.

To construct the recombinant virus rHVT-VP2, NHEJ-CRISPR/Cas9 gene-editing technology was used (Figure 1). Primary CEFs (4 × 10^5^) were seeded into 24-well plates two days before transfection, and 0.25 μg of PX459-UL45/46-gRNA, 0.25 μg of PX459-sgA-gRNA, and 0.5 μg of donor plasmid pGEM-sgA-LoxP-PacI-GFP-PacI-LoxP-SfiI-VP2-SfiI-sgA were transfected into CEFs using Lipofectamine 3000^®^ (Invitrogen, Carlsbad, CA, USA) according to the manufacture’s protocol. Then, 24 h after transfection, the cells were treated with puromycin for three days and then infected with HVT at a multiplicity of infection (MOI) value of 0.01 plaque-forming units (pfu)/cell. Three days after infection, half of the cells were used for PCR identification to determine if gene editing had occurred, and the other half were re-seeded in new CEFs for fluorescent plaque screening and purification (Figure 2).

### 2.4. Stability

The recombinant virus rHVT-VP2 was grown sequentially in CEFs for 10 passages, and the GFP-VP2 gene was examined via PCR using a DNA sample extracted from every 5 passages.

### 2.5. Vaccination and Challenge Experiment

All experimental and animal management procedures were undertaken following the requirements of the National Association of Laboratory Animal Care and the Ethics Committee of Jeonbuk National University. The animal experiments were approved by the Ethics Committee of Jeonbuk National University (approval number: NON2023-009).

To test the protective efficacy of vaccination with the recombinant virus rHVT-VP2, 41 one-day-old SPF chickens were distributed into 5 groups (A–C, positive control (PC), and negative control (NC). Group A (*n* = 10), Group B (*n* = 5), and Group C (*n* = 10) were vaccinated with rHVT-VP2, HVT, and commercial HVT-IBD vector vaccine (vaccine A), respectively, via the subcutaneous route under the skin of the neck with 6000 PFU in a 200 µL volume. The PC (*n* = 8) and NC (*n* = 8) groups were immunized with PBS in the same way. At 21 days post-vaccination, Groups A, B, and C and the PC group were challenged intraocularly with 200 µL of 1 × 10^7^ EID_50_ of A21-ETC-014 CE3 (G2d).

### 2.6. Serology

Blood samples were collected from each group at 7, 14, and 21 days post-vaccination. The samples were placed at 56 °C for 30 min and then centrifuged at 3000 rpm for 15 min to separate the sera. IBD-antibody titers were measured using commercial VDPro^®^ IBDV AB ELISA kit (Median Diagnostics, Chuncheon-si, Korea). According to the manufacturer’s instructions, serum samples with an ELISA antibody titer >750 were considered positive.

### 2.7. Clinical Signs, Mortality, and Postmortem Lesions

The birds were checked daily for clinical signs within a week after the challenge. At 7 days post-infection (dpi), all surviving birds were subjected to autopsy, and the gross lesions were examined.

### 2.8. Bursa-to-Body-Weight Index and Spleen Index

The bursa weight, spleen weight, and body weight were recorded for each bird. The b/B ratio was calculated using the following formula: bursa weight (grams)/body weight (grams) × 1000. The BBIX was calculated as follows: b/B ratio in the infected group/b/B ratio in the control group. A BBIX value less than 0.7 was considered indicative of atrophy. The s/B ratio was calculated as follows: spleen weight (grams)/body weight (grams) × 1000.

### 2.9. Statistical Analysis

Statistical analysis was performed using SPSS version 21.0 (SPSS Inc., Chicago, IL, USA). The data were analyzed using one-way ANOVA. Differences were considered statistically significant at * *p* < 0.05, ** *p* < 0.01, and *** *p* < 0.001.

## 3. Results

### 3.1. Identification of PX459-UL45/46-gRNA and PX459-SGA-gRNA

Using the PX459-UL45/46-gRNA plasmid as a template, a PCR was performed using the primers U6 forward and UL45/46-gRNA-R, and the target band of 98 bp was amplified (Supplementary Figure S1A). The sequencing results also indicated that PX459-UL45/46-gRNA was successfully constructed (Supplementary Figure S2A). Using PX459-sgA-gRNA as a template, a PCR was performed using the primers U6 forward and sgA-gRNA-R, and the target band of 98 bp was amplified (Supplementary Figure S1B). The sequencing results also indicated that PX459-sgA-gRNA was successfully constructed (Supplementary Figure S2B).

### 3.2. Generation of Recombinant rHVT-VP2

**Figure 3:**
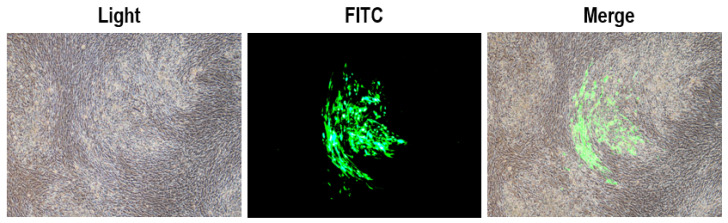
Plaques expressing green fluorescence were observed during the screening of rHVT-VP2.

To generate the recombinant virus rHVT-VP2, CEFs were co-transfected with PX459-UL45/46-gRNA, PX459-sgA-gRNA, and donor plasmid pGEM-sgA-LoxP-PacI-GFP-PacI-LoxP-SfiI-VP2-SfiI-sgA. At 24 h post-transfection, the cells were treated with puromycin for three days and then infected with HVT at an MOI of 0.01. At three dpi, fluorescent plaques expressing GFP appeared, indicating that the donor sequence GFP-VP2 had been successfully inserted into the HVT genome (Figure 3). Four pairs of primers were used for PCR identification. The primers UL45-F/VP2-R and G2d-3F/UL45/46-R were used to identify viruses inserted in the forward orientation (Supplementary Figure S3A), and the target bands were 2633 bp and 548 bp, respectively. The primers UL45-F/G2d-3F and VP2-R/UL45/46-R were used to identify viruses inserted in the reverse orientation (Supplementary Figure S3B), and the target bands were 589 bp and 2592 bp, respectively.

### 3.3. Purification of Recombinant rHVT-VP2

After three rounds of purification, the recombinant virus rHVT-VP2 was identified using PCR. The primer G2d-3F/UL45/46-R was used to identify the virus inserted in the forward orientation. The target band was 548 bp. The primer UL45-F/G2d-3F was used to identify the virus inserted in the reverse orientation. The target band was 589 bp. The primer UL45/46-F/UL45/46-R was used to detect the presence of HVT virus. The target band was 233 bp. The results showed that GFP-VP2 was inserted in the forward direction in the finally purified recombinant virus (Supplementary Figure S4).

### 3.4. Stability

The genetic stability of the GFP-VP2 gene was measured by passing rHVT-VP2 sequentially in CEFs for 10 passages. After every five passages, the viral DNA was extracted and analyzed using PCR. The PCR results showed that the target band was amplified from the 5th and 10th passage of the rHVT-VP2 DNA samples. These results indicated the stable integration of the GFP-VP2 gene in the UL45/46 locus of the HVT genome even after 10 passages.

### 3.5. Humoral Immune Response

**Figure 4:**
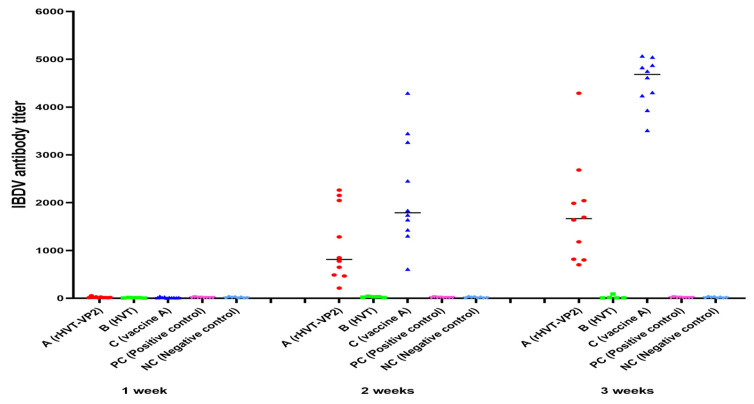
Detection of IBDV antibody from immunized chickens using ELISA.

To assess the immunogenicity of rHVT-VP2 in chickens, sera were collected weekly and checked using ELISA after vaccination. According to the manufacturer’s instructions, immune status was considered positive if the ELISA titer was above 750. Two weeks after immunization, the seropositivity rates of Groups A (rHVT-VP2), B (HVT), and C (vaccine A) were 50%, 0%, and 90%, respectively. The antibody titers of Groups A, B, and C were 952 ± 797, 28 ± 10, 2202 ± 1141, respectively. After three weeks, the seropositivity rates of Groups A, B, and C were 90%, 0%, and 100%, respectively, and the mean antibody titers were 1544 ± 1137, 24 ± 36, and 4517 ± 510, respectively. The average antibody level induced by rHVT-VP2 was higher than 750. These results suggest that although it induced lower levels of antibodies than the commercial vaccine A, rHVT-VP2 can induce high levels of humoral immunity (Figure 4).

### 3.6. Clinical Signs, Mortality, Pathology, and Virus Detection

No clinical signs or mortality were recorded in any groups during the 7-day observation period after the challenge. All the chickens were necropsied at 7 dpi. The NC group did not show any gross lesions. In the PC group, one of the eight chickens showed hemorrhaging of the thigh muscles. The main gross lesions of the chickens were atrophies of the bursae (Table 2). In the PC group and Group B (HVT), all the birds showed bursa atrophy. In Group C (vaccine A), 60% (6/10) of chickens showed bursa atrophy. However, none of the chickens in Group A (rHVT-VP2) showed bursa atrophy (0/10). The results for the b/B ratio and BBIX were also consistent with those for bursa lesions. The b/B ratios of the NC group and Group A (rHVT-VP2) were 5.02 ± 0.83 and 4.99 ± 0.54, respectively, with no statistically significant differences (*p* > 0.05). However, the b/B ratios of the PC group and Group B (HVT) were significantly lower at 1.61 ± 0.29 and 1.33 ± 0.23, respectively, with a statistically significant difference (*p* < 0.05). The b/B ratio of Group C (vaccine A, 3.20 ± 1.66) was also lower than that of the NC group (5.02 ± 0.83).

Regarding the BBIX value, Group A (rHVT-VP2) showed the highest value (0.99 ± 0.11). The PC group and group B (HVT) showed the lowest values (0.32 ± 0.06 and 0.27 ± 0.05, respectively), and that of Group C (vaccine A) was in between (0.64 ± 0.33). Regarding the s/B ratio, there was no significant difference between the NC group, Group A (rHVT-VP2), and Group C (vaccine A), but the values in the PC group and Group B (HVT) were significantly increased (*p* < 0.05), indicating that the spleens of chickens in these two groups underwent a certain degree of swelling. Since the main damage caused by IBDV (G2d) to chickens is bursa atrophy, we calculated the protection index (PI) based on the atrophy rate of the bursa. The PIs of Group A (rHVT-VP2), Group C (vaccine A), and Group B (HVT) were 100%, 40%, and 0%, respectively. In addition, the bursae were also tested for IBDV using PCR. One test was conducted to determine whether IBDV was present, and the other was performed to confirm whether the virus was the strain used for the challenge or the vaccine. The results showed that no IBDV was detected in Group A (rHVT-VP2) (0/10), and only the challenge strain of IBDV (G2d) was detected in Groups B and C and the PC group, with detection rates of 80% (4/5), 40% (4/10), and 100% (8/8), respectively. These results indicated that rHVT-VP2 could provide 100% protection against IBDV G2d infection, while the commercial vaccine A only provided partial protection (Supplementary Figure S5).

## 4. Discussion

IBD is an economically important disease in many countries because of its potential impact on the poultry industry [1]. Novel variant IBDV is widespread and has become the most prevalent genotype of IBDV in poultry in Korea and other Asian countries [4,5,6,7]. In addition, novel variant IBDV has been continually spreading in vaccinated chicken flocks and is significantly different from vvIBDV; therefore, novel variant IBDV could overcome the immunoprotection afforded by the current commercial vaccines used in the chicken industry [7]. The present study describes the development of an HVT-vectored vaccine harboring the VP2 gene from novel variant IBDV G2d that can effectively protect against novel variant IBDV infection.

Currently, vaccination remains the most effective strategy for preventing and controlling infectious bursal disease virus (IBDV) infection in chickens. Various types of commercial vaccines are available, including inactivated and live attenuated vaccines, immune complex vaccines, subunit vaccines, and live viral vector vaccines. These vaccines offer protection against both classic and very virulent strains of IBDV [18]. Several viral vectors, such as fowl pox virus (FPV), adenovirus, infectious laryngotracheitis virus (ILTV), and Marek’s disease virus (MDV), have been extensively utilized in the development of poultry viral vaccines. Recently, recombinant herpesvirus of turkey (rHVT)-IBD vaccines have gained prominence for their superior efficacy, particularly in progeny application. These vaccines are engineered to express only the VP2 gene from classic-type IBDV strains, thereby ensuring the expression of protective immunogens that induce immunity against IBDV VP2 [8,9]. In agreement with the findings of previous studies, HVT-vectored IBDV VP2 vaccines can induce serum antibodies against IBDV and provide efficient protection against the same genotype of IBDV infection [18]. In the present study, three weeks after immunization, 90% of the chickens immunized with rHVT-VP2 produced high levels of humoral antibodies (1544 ± 1137) against IBDV. As with the results from previous studies, serum antibodies correlate with protection against IBDV challenge; all the chickens immunized with rHVT-VP2 did not show atrophy and pathological lesions of the bursae after an IBDV challenge.

As anticipated, recombinant HVT vector vaccines require longer periods to induce immunity compared to live attenuated vaccines [1]. A three-week interval provides adequate protection against antigenic variant IBDV infection in SPF chickens immunized with rHVT-VP2. Although HVT vector vaccines are well known to be safe and poorly sensitive to maternally derived antibody (MDA) interference in commercial chickens, more studies are needed to determine the protective efficacy of rHVT-VP2 for commercial chickens with MDA in the field. Notably, lower antibody titers were observed in the rHVT-VP2-immunized chickens than in the control vaccine-A-immunized chickens in this study. Antigenic variation of the VP2 gene from IBDV G2d compared to previous classic IBDV strains and mismatches in the antigens from classic IBDV strains coated on commercial ELISA kits are likely contributing factors. This result is consistent with previous studies indicating that novel variant G2d strains exhibit high antigenic differences and lack cross-neutralizing activity with classic IBDV strains [7]. Despite lower serum antibody titers detected using non-antigen-matched ELISA kits, the chicken challenge results indicate that the antibody titers may still be sufficient to neutralize the challenged virus and prevent its replication. It would also be of interest to update the antigen-matched ELISA method, as ELISA is a routine method of monitoring antibody levels in IBDV-immunized chickens, particularly when new IBDV vaccines against G2d are used to control the strain thereafter.

Reduced viral transmission is an important indicator of protection for determining IBDV vaccine efficacy [18]. In this study, no IBDV was detected in the chickens immunized with rHVT-VP2 after a challenge with novel variant IBDV. Thus, these results indicate that novel variant IBDV replication could be blocked completely in chickens after immunization with this newly developed rHVT-VP2 vaccine. In contrast, IBDV could be detected in the chickens immunized with commercial vaccine A. These results are in agreement with those of previous studies on the current commercial vaccines against vvIBDV, which were unable to provide complete protection against novel variant IBDV viruses in chickens [7]. In addition, the fact that novel variant IBDV was detected in 40% of chickens immunized with vaccine A is correlated with the rapidly increasing prevalence of novel variant IBDV in immunized chickens in the field [4]. The newly developed rHVT-VP2 vaccine presented in this study can provide protection against novel variant IBDV and minimize IBDV shedding; thus, this rHVT-VP2 vaccine could be an ideal live vaccine candidate for controlling the currently circulating novel variant IBDV infection.

Conventional recombination techniques have been used to develop the existing recombinant HVT-IBDV vaccines. However, these techniques are time consuming and laborious [19,20]. In the present study, we used the simple and effective CRISPR/Cas9-based gene-editing platform to generate a new recombinant IBDV vaccine against the current novel variant IBDV. Innovative CRISPR/Cas9-based gene editing has previously been successfully applied for editing the genomes [11,21,22]. Based on the success of these studies, we used the CRISPR/Cas9 technique to generate a new HVT recombinant vaccine that expresses the IBDV VP2 protein from genotype G2d, a current specific genotype widely circulating in Asian countries [4,5,6,7]. Moreover, we employed a more efficient non-homologous end-joining (NHEJ) pathway to introduce the VP2 gene into the HVT genome. Double-strand breaks (DSBs) produced by Cas9 are repaired primarily through two repair pathways: NHEJ and homology-directed repair (HDR) [23]. NHEJ can occur at any phase of the cell cycle, whereas HDR can only occur during the S and G2 phases; the NHEJ pathway is able to repair DNA within 30 min, whereas HDR may require a 7 h delay to complete the process [24,25,26]. Furthermore, the donor plasmid does not need to be added with homology arms, and its production process is shorter [24,25,26]. Therefore, as in previous studies, NHEJ-CRISPR/Cas9 is considered to offer significant convenience and efficiency for the knock-in of foreign genes and for generating recombinant viruses. More importantly, this approach could also be used to edit multiple genes at once [27]. Therefore, the use of this state-of-art technology could greatly accelerate multivalent vaccine development [13,21].

It is worth noting that unintended off-target edits might confer risks in the application of the CRISPR/Cas9 system to gene editing. Although gRNA design algorithms are under continual refinement, off-target activity can be unavoidable when the gRNA window is restricted to a narrow genomic location [28]. Further studies are required to confirm the off-target mutations in rHVT-VP2 generated by the CRISPR/Cas9 system, such as those involving full-genome sequencing.

HVTs, which belong to Meleagrid herpesvirus 1, have been widely used for more than 40 years in vaccines against Marek’s disease (MD) [29]. HVT is an efficient delivery system for several immunogenic genes that help to control multiple poultry diseases [8,10,20]. HVT binds tightly to host cells and spreads from cell to cell. Therefore, it still shows good efficacy in the presence of maternal antibodies [9]. In addition, HVT is a cell-binding virus that binds tightly to host cells; thus, foreign genes can be continuously expressed in host cells to provide life-long immunity in chickens. In addition, the HVT genome is large enough to accommodate the insertion of multiple foreign genes [30]. In this study, we developed a recombinant HVT vector vaccine expressing the IBDV VP2 antigen from genotype G2d, showing effective protection against novel variant IBDV in SPF chickens. The HVT vector vaccine can induce both humoral and cell-mediated immunity and provide protection in the presence of maternal immunity [8]. Thus, the candidate vector vaccine has important value and the potential for clinical application in the poultry industry to control IBDV infection in the field. Furthermore, HVT vector can be detected in chickens throughout the laying period of broiler breeders. HVT vector vaccines have the capability to continuously stimulate the immune system, thereby maintaining circulating antibody levels against IBDV in breeder chickens [8,9]. The rHVT-VP2 vaccine developed in this study offers the advantage of providing protection with a single vaccination throughout the lives of breeder chickens.

## 5. Conclusions

In conclusion, the recombinant virus rHVT-VP2 was successfully constructed using NHEJ-CRISPR/Cas9 gene-editing technology. The vaccination of SPF chickens with rHVT-VP2 produced specific antibodies against IBDV and resistance to a challenge with IBDV G2d strains. Therefore, rHVT-VP2 could be used as a vaccine candidate against IBDV G2d in chickens in the field. In addition, our results lay the foundation for the construction of a multivalent HVT live vector vaccine through the application of the CRISPR/Cas9 system platform. Overall, the successful construction of the rHVT-VP2 vaccine candidate and the potential of the CRISPR/Cas9 system platform offer promising avenues for combating pathogens in poultry. Further research and development efforts, including rigorous testing and field trials, will be necessary to fully evaluate the safety, efficacy, and practicality of these vaccine candidates. Nonetheless, the utilization of advanced gene-editing technologies such as CRISPR/Cas9 holds great potential for revolutionizing the field of poultry disease prevention and improving the overall health and welfare of commercial chicken populations.

## Figures and Tables

**Table 1:** List of primers used in this study.

Primer	Sequence (5′-3′)	Reference
UL45/46-gRNA-F	CACCGAAAACACAGTAACCGTTAG	[11]
UL45/46-gRNA-R	AAACCTAACGGTTACTGTGTTTTC	
sg-A-gRNA-F	CACCGAGATCGAGTGCCGCATCAC	
sg-A-gRNA-R	AAACGTGATGCGGCACTCGATCTC	
U6 forward	GACTATCATATGCTTACCGT	
UL45-F	TGTCGGCAGACTGTCCTGTA	
VP2-R	GTGCATGACCGTGCTGATTC	
G2d-3F	CGTCTTGGCATCAAGACCGT	
UL45/46-F	GATGCCCGCGTGTATCTTCA	
UL45/46-R	ACGTAGGCTGAAAGTGTCCAG	

**Table 2:** Protective efficacy against IBDV (G2d) challenge.

Group	7 Days Post-Challenge							
	Mortality	Gross Lesion ^a^	Bursa Lesion ^b^	b/B Ratio ^c^	BBIX ^d^ (Atrophy, %)	s/B Ratio ^e^	Virus Detection in Bursa	PI ^f^
A (rHVT-VP2)	0/10 (0%)	0/10 (0%)	0/10 (0%)	4.99 ± 0.54	0.99 ± 0.11 (0/10, 0%)	2.36 ± 0.61	0/10 (0%)	100%
B (HVT)	0/5 (0%)	0/5 (0%)	5/5 (100%)	1.33 ± 0.23	0.27 ± 0.05 (5/5, 100%)	3.12 ± 0.46	4/5 (80%)	0%
C (vaccine A)	0/10 (0%)	0/10 (0%)	6/10 (60%)	3.20 ± 1.66	0.64 ± 0.33 (6/10, 60%)	2.58 ± 0.55	4/10 (40%)	40%
PC (Positive control)	0/8 (0%)	1/8 (12.5%)	8/8 (100%)	1.61 ± 0.29	0.32 ± 0.06 (8/8, 100%)	3.29 ± 0.39	8/8 (100%)	-
NC (Negative control)	0/8 (0%)	0/8 (0%)	0/8 (0%)	5.02 ± 0.83	-	1.94 ± 0.36	0/8 (0%)	-

^a^ Gross lesion: leg muscle hemorrhage, kidney swelling and hemorrhage, and proventriculus hemorrhage. ^b^ Bursa lesion: bursa hemorrhage, inflammation, and atrophy. ^c^ b/B ratio = bursa weight/body weight × 1000. ^d^ BBIX = (b/B ratio in the infected group)/(b/B ratio in the control group). A BBIX value less than 0.7 was considered to indicate atrophy. ^e^ s/B ratio = spleen weight/body weight × 1000. ^f^ PI: protection index = 100% − bursa atrophy rate of the vaccinated group/bursa atrophy rate of the positive group.

## Data Availability

The data presented in this study are available from the corresponding author on reasonable request.

## Supplementary Materials

The following supporting information can be downloaded at https://www.mdpi.com/article/10.3390/vaccines12030226/s1. Figure S1: Identification of Cas9/gRNA using PCR. (A) Identification of PX459-UL45/46-gRNA via PCR with primers U6 forward and UL45/46-gRNA-R. (B) Identification of PX459-sgA-gRNA via PCR with primers U6 forward and sgA-gRNA-R. Figure S2: Sequencing result of Cas9/gRNA expression plasmids. (A) Sequencing result of PX459-UL45/46-gRNA with primer U6 forward. (B) Sequencing result of PX459-sgA-gRNA with primer U6 forward. Figure S3: PCR identification of recombinant virus rHVT-VP2 with GFP-VP2 forward and reverse insertion. (A) The primers UL45-F/VP2-R and G2d-3F/UL45/46-R were used to identify viruses inserted in the forward orientation, and the target bands were 2633 bp and 548 bp, respectively. (B) The primers UL45-F/G2d-3F and VP2-R/UL45/46-R were used to identify viruses inserted in the reverse orientation, and the target bands were 589 bp and 2592 bp, respectively. Figure S4: Identification of purified recombinant virus rHVT-VP2 via PCR. The primer G2d-3F/UL45/46-R was used to identify the virus inserted in the forward orientation. The primersUL45-F/G2d-3F was used to identify the virus inserted in the reverse orientation. The primer UL45/46-F/UL45/46-R was used to detect the presence of HVT. Figure S5: The bursa of Fabricius recovered from the chickens at day 7 post-challenge with the IBDV (G2d) strain.
