# Supplementary figures and images for: Development of a Highly Efficient CRISPR/Cas9-Mediated Herpesvirus of Turkey-Based Vaccine against Novel Variant Infectious Bursal Disease Virus
Source: Vaccines (Basel). 2024 Feb 23;12(3):226. doi: 10.3390/vaccines12030226 (PMC10974780; doi:10.3390/vaccines12030226)

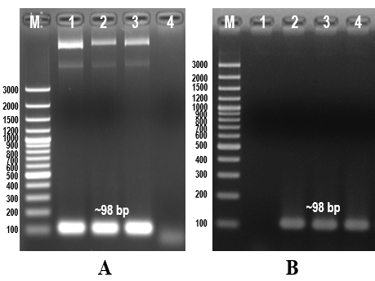

Supplement: Supplementary file 1 [file vaccines-12-00226-s001.zip › Figure S1.tif]

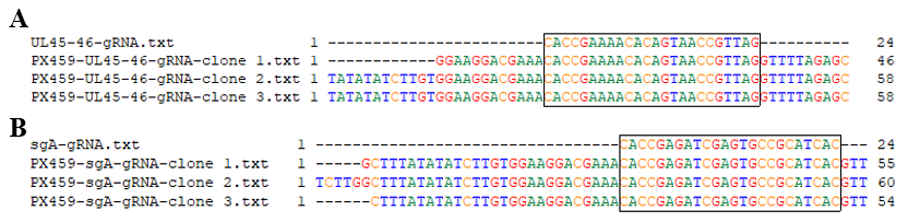

Supplement: Supplementary file 1 [file vaccines-12-00226-s001.zip › Figure S2.tif]

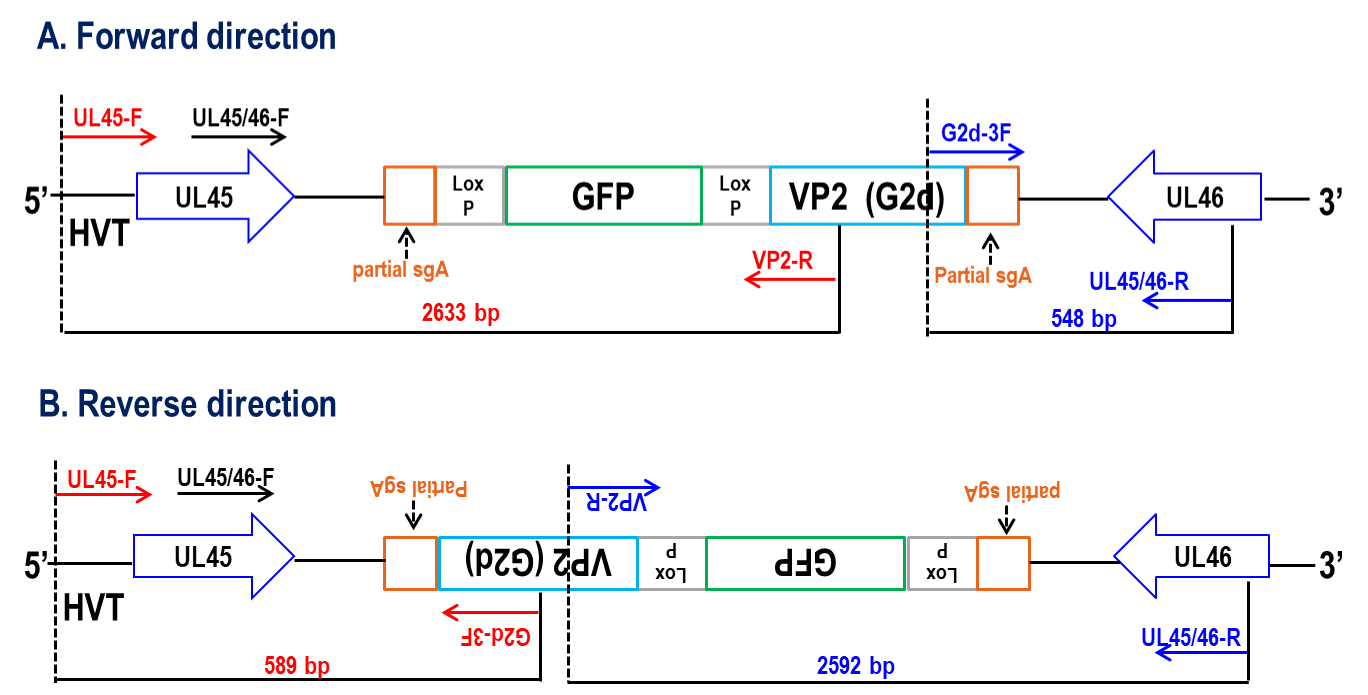

Supplement: Supplementary file 1 [file vaccines-12-00226-s001.zip › Figure S3 .tif]

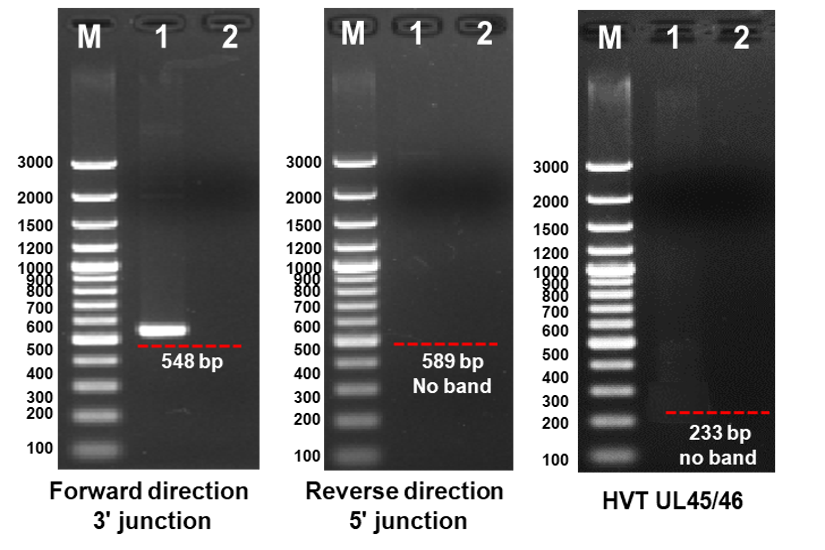

Supplement: Supplementary file 1 [file vaccines-12-00226-s001.zip › Figure S4 .tif]

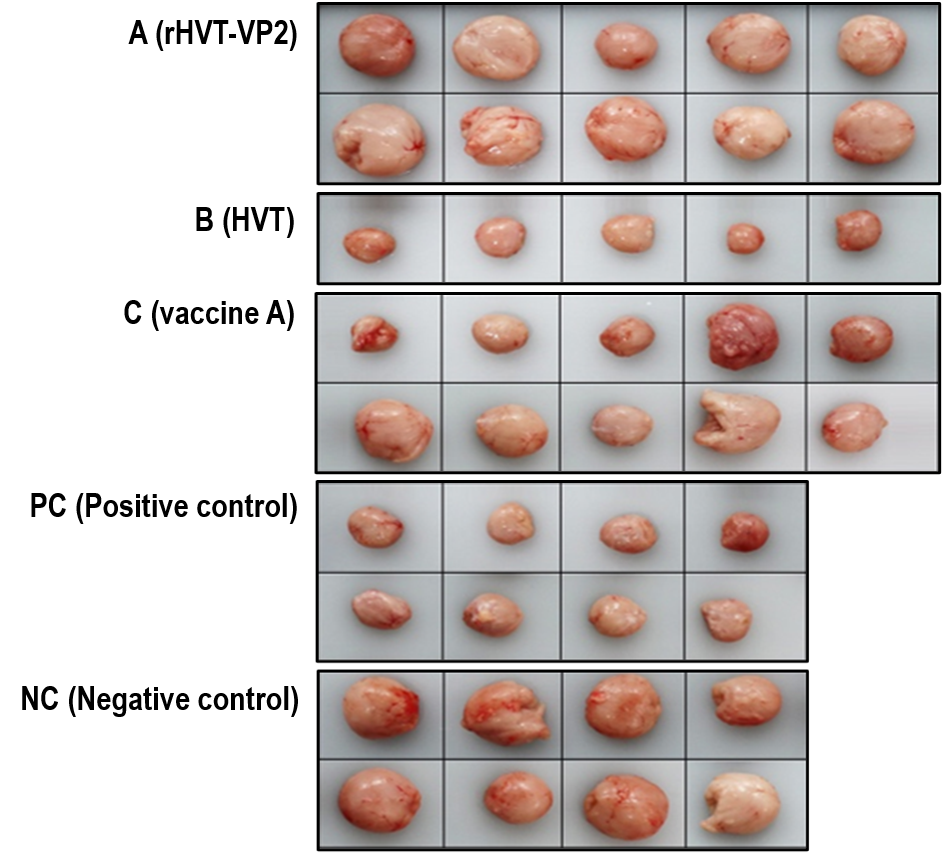

Supplement: Supplementary file 1 [file vaccines-12-00226-s001.zip › Figure S5 .tif]
